# Self-wavelength shifting in two-dimensional perovskite for sensitive and fast gamma-ray detection

**DOI:** 10.1038/s41467-023-38545-y

**Published:** 2023-05-17

**Authors:** Tong Jin, Zheng Liu, Jiajun Luo, Jun-Hui Yuan, Hanqi Wang, Zuoxiang Xie, Weicheng Pan, Haodi Wu, Kan-Hao Xue, Linyue Liu, Zhanli Hu, Zhiping Zheng, Jiang Tang, Guangda Niu

**Affiliations:** 1 Wuhan National Laboratory for Optoelectronics and School of Optical and Electronic Information, Huazhong University of Science and Technology, Wuhan, 430074 China; 2 Shenzhen Institute of Advanced Technology, Chinese Academy of Sciences, Shenzhen, 518055 China; 3 State Key Laboratory of Intense Pulsed Radiation Simulation and Effect, Northwest Institute of Nuclear Technology, Xi’an, 710024 China; 4 Optics Valley Laboratory, Hubei, 430074 China

**Keywords:** Materials for optics, X-rays

## Abstract

Lead halide perovskites have recently emerged as promising X/γ-ray scintillators. However, the small Stokes shift of exciton luminescence in perovskite scintillators creates problems for the light extraction efficiency and severely impedes their applications in hard X/γ-ray detection. Dopants have been used to shift the emission wavelength, but the radioluminescence lifetime has also been unwantedly extended. Herein, we demonstrate the intrinsic strain in 2D perovskite crystals as a general phenomenon, which could be utilized as self-wavelength shifting to reduce the self-absorption effect without sacrificing the radiation response speed. Furthermore, we successfully demonstrated the first imaging reconstruction by perovskites for application of positron emission tomography. The coincidence time resolution for the optimized perovskite single crystals (4 × 4 × 0.8 mm^3^) reached 119 ± 3 ps. This work provides a new paradigm for suppressing the self-absorption effect in scintillators and may facilitate the application of perovskite scintillators in practical hard X/γ-ray detections.

## Abstract (web summary)

Efficient light extraction in perovskite X/γ-ray scintillators is hindered by the small Stokes shift of exciton luminescence. Here, Jin et al. exploit the intrinsic strain in 2D perovskite as “self-wavelength shifting” to reduce the self-absorption effect without sacrificing the device response speed.

## Introduction

X-ray and γ-ray detections play vital roles in various applications, including medical imaging, non-destructive inspection, security checking, and radioisotope identification^1–3^. Scintillation detection dominates the high-energy radiation detection market considering its industrial feasibility with low cost, rich choices for customization, and flexible combination with commercially mature sensors (Si photodiode, Si photomultiplier (Si-PM), and photomultiplier tube (PMT))^4^. Conventional scintillators, such as CsI: Tl and Lu_1.9_Y_0.1_SiO_5_ (LYSO): Ce, are usually synthesized by the Czochralski and Bridgman method with ultrahigh processing temperature greater than 1700 °C, which is neither cost-effective nor user-friendly^5,6^. The recently reported lead halide perovskite scintillators have demonstrated excellent properties for soft X-ray detection (<70 keV) because of the convenient solution synthesis process, considerable stopping power, low detection limit, and short radioluminescence (RL) lifetime^7–9^. For example, CsPbBr_3_ nanocrystals enable the fabrication of flexible and sensitive X-ray detectors with a detection limit of 13 nGy_air_ s^−1^ with the X-ray source operating at a voltage of 50 kV^7^. On the other hand, practical applications require hard X-rays and even γ-rays for high penetration capability, such as computed tomography (CT) imaging (80–120 keV)^10^, security checking (>160 keV)^11^, and positron emission tomography (PET, 511 keV)^12^. For hard X-rays and γ-rays, the corresponding linear attenuation coefficients for scintillators are much smaller than that of soft X-rays, and the typical thickness of lead halide perovskite scintillators (4–120 μm) is obviously inadequate. Hence, much thicker crystals should be used to retain the attenuation efficiency.

Nevertheless, the criticized small-to-vanishing Stokes shift of exciton luminescence in perovskite scintillators creates severe self-absorption problems, especially in large thicknesses (mm-cm)^13^. Endeavors have been devoted to addressing the self-absorption issues of perovskite scintillators. Available strategies include (1) using self-trapped exciton luminescence^14–19^ and (2) adding dopants (Mn^2+^, Lu^3+^ and organic chromophores with thermally activated delayed fluorescence) to shift the emission wavelength^20,21^. Unfortunately, although the self-absorption could be suppressed, the RL lifetime is severely extended from hundreds of nanoseconds to milliseconds, which strongly impedes their deployment in CT imaging and time-of-flight γ-ray detections. The long lifetime is due to the forbidden transition character for the emitters, including spin-forbidden triplet transition for self-trapped exciton and organic chromophores, parity-forbidden *d-d* transition for Mn^2+^_,_ and *f-f* transition for Lu^3+^. Hence, the development of halide scintillators with suppressed self-absorption and retained fast luminescence decay remains a grand challenge.

In this work, we posit the intrinsic strain in 2D perovskite crystals as a general phenomenon, which could be utilized to suppress the self-absorption effect without sacrificing the luminescence lifetime. The strain effect has been widely found in perovskite single crystals and thin films. For example, Chen and co-workers have investigated the emission difference between the surface and bulk of 2D perovskites in the context of crystal distortion^22^. However, the origin of the structural distortion is still not comprehensive, and its possible benefit in scintillators has not been studied. To this end, a group of 2D perovskite single crystals (PEA_2_PbBr_4_, BA_2_PbBr_4_, and PEA_2_PbI_4_, where PEA and BA stand for phenylethylamine and butylammonium, respectively) has been investigated for their good quantum and dielectric confinement. The grazing-incidence wide-angle X-ray scattering (GIWAXS) exhibited a strained structure on the crystal surface compared to the inside. The X-ray photoelectron spectroscopy and theoretical calculations indicate the lack of surface amines as the structural origin of the induced strain. As a result, the band gap of the crystal surface is enlarged, while the fast radiative-recombination channel is retained. The transient photoluminescence spectroscopy clearly revealed the rapid energy transfer process from surface to inside bulks, establishing the foundation for simultaneously achieving self-wavelength shifting and fast RL lifetime. Furthermore, we successfully applied the SWS scintillators in high energy γ-ray detection and demonstrated the imaging reconstruction in PET, which is the first time ever reported for perovskite scintillators.

## Results

Firstly, we quantitatively analyzed the desired thickness of perovskite toward X/γ-ray. CsPbBr_3_ is studied as a standard model, and definition of absorption efficiency and optical efficiency were given in Supplementary Note 1. The absorption coefficient of CsPbBr_3_ to X/γ photons is shown in Supplementary Fig. 1a, with values of 36.22 cm^−1^, 2.46 cm^−1^, and 0.45 cm^−1^ for 50 keV, 200 keV, and 600 keV photons, respectively, which indicates that the attenuation coefficients of hard X/γ-rays for scintillators are much smaller than that of soft X/γ-rays. Absorption efficiency as a function of thickness for different energy photons is shown in Supplementary Fig. 1b, which reveals that a much thicker scintillator is required to improve the absorption efficiency of high-energy photons.

Subsequently, we plot the optical efficiency of visible light photons with energies higher than the edge of the absorption band (i.e., visible light photons prone to self-absorption) as a function of thickness. Due to their considerable absorption coefficients (generally in the range of 10^3^−10^5^ cm^−1^), it is difficult for them to pass through such a thick material. As shown in Supplementary Fig. 1c, the optical efficiency decreases dramatically as the thickness increases. When the thickness exceeds 0.94 cm, the optical efficiency of visible light photons prone to self-absorption drops to less than 1%. As a result, it is difficult for these visible light photons prone to self-absorption to penetrate such thick crystals and reach visible-light detectors, which results in a decrease in optical transparency. Here the optical transparency is defined as

1 $${\eta }_{transparency}=1-{\eta }_{self-absorption}=1-\frac{\int PL(\lambda )\times Abs(\lambda )d\lambda }{\int PL(\lambda )d\lambda }$$

where the fraction term represents the normalized self-absorption efficiency *η*_*self-absorption*_, *λ* is the wavelength, *PL(λ)* is the normalized photoluminescence (PL) spectrum, and *Abs(λ)* is the normalized absorption spectrum. High optical transparency represents a low self-absorption effect and high light extraction efficiency.

**Fig. 1:**
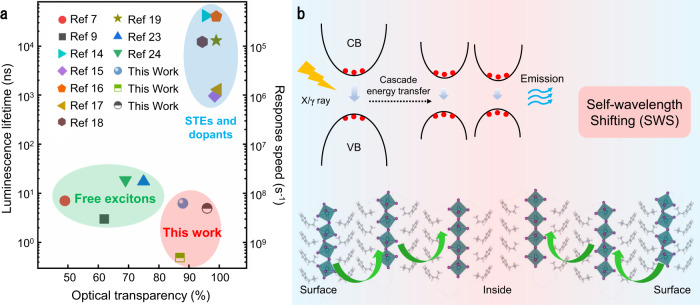
Perovskite scintillator performance summary and self-wavelength shifting schematic diagram. **a** The scatter diagram of luminescence lifetime versus optical transparency for the reported perovskite-based scintillators. **b** The schematic illustration of the energy level and exciton funneling process of the self-wavelength shifting in two-dimensional perovskites.

To better illustrate the field status of perovskite scintillators, we plot the scatter diagram of luminescence lifetime (the reciprocal of response speed) versus optical transparency (Fig. 1a). The luminescence lifetime and optical transparency for different types of perovskite scintillators are summarized in Supplementary Table 1. Clearly, the nanocrystals, like CsPbBr_3_ quantum dots (QD), MAPbBr_3_ QDs, FAPbBr_3_ QDs, and CsPbBr_3_@Cs_4_PbBr_6_, exhibit short luminescence lifetime but severely low optical transparency^7,9,23–25^. Self-trapped excitons and mixtures of perovskite with dopants show high optical transparency but relatively low response speed^14–17,20,21^. Ideally, perfect scintillators are expected to reside at the bottom right corner of Fig. 1a with high response speed and high light extraction efficiency.

The concept of SWS is illustrated in Fig. 1b. The bandgap gradually decreases from the crystal surface to the inside bulks, which is a crucial point and will be elaborated on later. Then the X/γ ray excited excitons could follow a cascade energy transfer behavior into the lowest energy states from either the route of photo recycling effect, charge energy transfer or probably Förster resonant energy transfer (FRET). The final emission, which is expected deep inside the crystal, could effectively pass through the neighboring and outer lead halide layers, which possess larger bandgaps.

To verify the correctness and universality of the method, we have grown three kinds of 2D perovskite single crystals (PEA_2_PbBr_4_, BA_2_PbBr_4_, and PEA_2_PbI_4_) with bright luminescence. The absorption coefficients for the 2D perovskites are compared with the traditional scintillators in Supplementary Fig. 2. Due to the presence of heavy element Pb, absorption coefficients of the three 2D perovskites for high-energy photons are much higher than that of stilbene. This ensures the effective absorption of high-energy photons. On this basis, we also obtained the absorption efficiency as a function of thickness in the three 2D perovskites for different photon energies in Supplementary Fig. 3 through the Beer-Lambert law. It should be noted that many previous works have reported the successful synthesis of 2D crystals, but the crystal size is generally too small (<2 mm), and the crystal transparency is insufficient to satisfy their application in X/γ-ray detection^26,27^. We used the seed-assisted volatile solvent to harvest large and high-quality single crystals (see details in Supplementary Fig. 4). The crystals were dried with nitrogen and stored in the glove box.

**Fig. 2:**
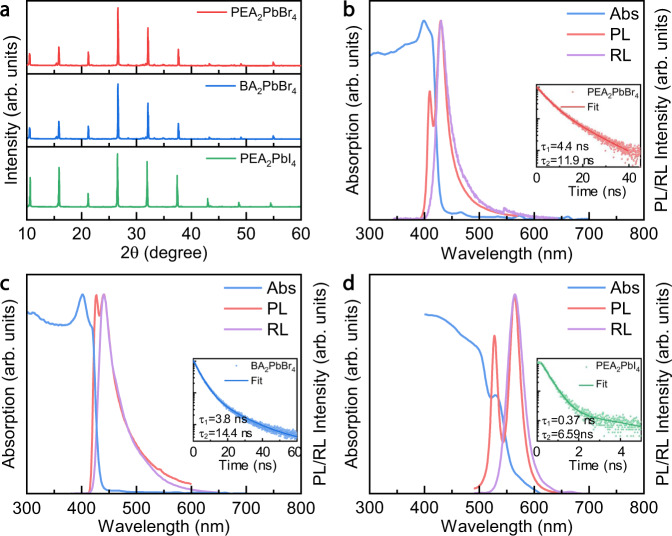
Comparison of the photoluminescence and radioluminescence spectra of two-dimensional perovskites. **a** Single-crystal XRD of PEA_2_PbBr_4_, BA_2_PbBr_4_, and PEA_2_PbI_4_ respectively. **b–d** The absorption (Abs), photoluminescence (PL), radioluminescence (RL) and time-dependent PL spectra of PEA_2_PbBr_4_ (**b**), BA_2_PbBr_4_ (**c**) and PEA_2_PbI_4_ (**d**).

Then PEA_2_PbBr_4_, BA_2_PbBr_4_, and PEA_2_PbI_4_, with crystal dimensions over 15 mm × 5 mm (Supplementary Fig. 5), were obtained. The thicknesses of PEA_2_PbBr_4_, BA_2_PbBr_4_ and PEA_2_PbI_4_ single crystals are 2.46 mm, 1.12 mm and 1.28 mm, respectively. The X-ray diffraction (XRD) patterns are shown in Fig. 2a. The periodically distributed diffraction peaks corresponding to (00 *l*) (*l* = 2, 4, 6 …) lattice planes indicate good crystallinity and orientation of the grown crystals. Their crystal structure is triclinic. The corresponding space groups are *P*$\bar{1}$, *P*1 and *P*1, and the crystal plane spacings are 0.831 nm, 0.831 nm, and 0.843 nm for PEA_2_PbBr_4_, BA_2_PbBr_4_, and PEA_2_PbI_4_, respectively, similar to that reported in the literature^28,29^.

We further investigated the optical performances of the three crystals. The absorption and PL curves under both UV light and X-rays are shown in Fig. 2b–d. The absorption edges are at 425 nm, 428 nm and 553 nm, corresponding to bandgaps of 2.92 eV, 2.90 eV and 2.24 eV for PEA_2_PbBr_4_, BA_2_PbBr_4_ and PEA_2_PbI_4_, respectively. It should be noted that there is a trailing of the luminescent peak at the long wavelength, which leads to the asymmetry in the shape of the spectrum. It is inferred that this phenomenon is related to the defect-induced band tail state^30^. For the RL peak, we took the opinion that the energy transfer process from the surface to the inner parts could produce a series of emission states, and this also contributes to the asymmetric shape. Interestingly, we could observe two emission peaks in the PL spectra under 320 nm excitation, i.e., 409 nm and 430 nm for PEA_2_PbBr_4_, 427 nm, and 441 nm for BA_2_PbBr_4_, 527 nm and 564 nm for PEA_2_PbI_4_. A similar phenomenon has been observed in previous reports^22^. The underlying mechanism is still under debate, and some speculations include the A-site cation stacking-induced strain effect or possible structural differences between the surface and bulk states. In order to understand the origin of the dual peaks, we changed the incident light direction during PL measurement (Supplementary Fig. 6). By changing the incident angle from 90^o^ to 45^o^, the proportion of the long-wavelength emission peak of PEA_2_PbBr_4_ drastically decreased and was mainly manifested as the short-wavelength emission peak. As the incident angle decreases, the PL from the surface layer dominates the PL spectra, which confirms the increased bandgap of the surface layer.

For RL spectra under X-ray excitation, in contrast, there is only one emission peak at the long wavelength, i.e., 430 nm for PEA_2_PbBr_4_, 440 nm for BA_2_PbBr_4_, and 565 nm for PEA_2_PbI_4_. It should be noted that the collected luminescence of PL and RL are from different directions, with the reflected luminescence collected in PL mode and transmitted luminescence in RL mode. Thereby, the RL spectra represent the luminescence spectra from the entire bulk crystals compared with the surface luminescence in PL. This also supports the process in Fig. 1b.

We also recorded the time-resolved photoluminescence (TRPL) spectra of the 2D crystals, as shown in the inset of Fig. 2b–d. The curves can be fitted in terms of biexponential decay, with τ_1_ = 4.4 ± 0.15 ns (75.1%), τ_2_ = 11.9 ± 1.54 ns (24.9%) for PEA_2_PbBr_4_, τ_1_ = 3.6 ± 0.03 ns (89.2 %), τ_2_ = 14.4 ± 0.69 ns (10.8%) for BA_2_PbBr_4_, and τ_1_ = 0.37 ± 0.01 ns (96.6%), τ_2_ = 6.58 ± 0.5 ns (3.4%) for PEA_2_PbI_4_. The average lifetimes are 6.27 ns, 4.94 ns, and 0.58 ns for PEA_2_PbBr_4_, BA_2_PbBr_4_, and PEA_2_PbI_4_, respectively. The short lifetime is possibly due to surface recombination, and the long lifetime component is attributed to bulk recombination. The fitting results are summarized in Supplementary Table 2.

**Fig. 3:**
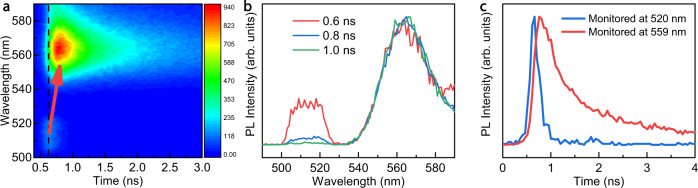
The energy transfer process between the surface and inside excitons of two-dimensional perovskite. **a** Time-resolved PL kinetics collected in the emission channels of 500–590 nm. The red arrow represents the Förster resonant energy transfer of photons from 520 nm to 559 nm. **b** Normalized PL spectra with the different time delays of 2D-perovskites. **c** Normalized PL decay curves monitored at 520 nm and 559 nm wavelength, respectively.

To gain the underlying mechanism and kinetic parameters between the surface and inside states, we collected the transient PL signals of these 2D perovskites in both wavelength and time domains (Fig. 3a). Here we mainly focus on PEA_2_PbI_4_ because of the convenient use of 470 nm excitation light, while the excitation of PEA_2_PbBr_4_ (430 nm) or BA_2_PbBr_4_ (440 nm) relies on UV light, which is beyond the capability of the transient PL facility. The transient PL profiles showed the initial emission from the surface (520 nm) and the emergence of inside emission (560 nm) after ~0.3 ns. Figure 3b also exhibits noticeable intensity changes of the two PL peaks. Figure 3c shows the time-dependent PL at two emission wavelengths. The fitted lifetimes are 0.14 ± 0.02 ns and 1.08 ± 0.02 ns for 520 nm and 559 nm, respectively. We extrapolated the rising time of 559 nm emission (Fig. 3c), which equals the time interval from 10% to 90% of the measured PL intensity. Indeed, the rising time of 559 nm emission (acceptor) is as short as 0.19 ± 0.03 ns, which is quite similar to the decay time (0.14 ± 0.02 ns) of 520 nm (donor). Such consistency confirms the presence of the FRET process that the surface excitons could non-radiatively transfer their energy to the inside excitons, and the energy transfer rate is ~10^10^ s^−1^, close to the previously reported value in Ruddlesden-Popper phase perovskites (2–7 × 10^10^ s^−1^)^31^. The kinetic parameter indicates the absence of photon recycling and charge energy transfer since in photon recycling, the transfer time could be as short as 6.7 ps within the given thickness of the crystal (2 mm/3 × 10^8^ m s^−1^), and the presence of long-chain amines between lead halide layers prevents the charge energy transfer.

**Fig. 4:**
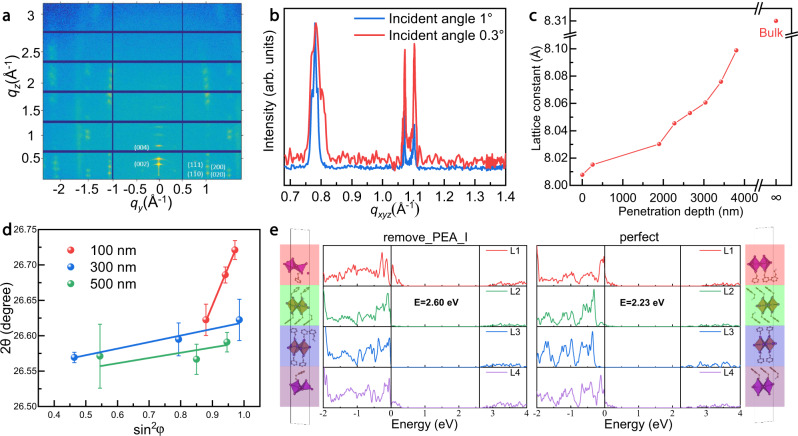
Structural and theoretical studies of the surface and inside bulks of the 2D-perovskite. **a** GIWAXS images of the surface structure (incident angle of 0.3°). **b** GIWAXS profiles of PEA_2_PbI_4_ with two incident angles. **c** The plot of lattice constant versus penetration depth. **d** Residual strain distribution in the depth of 100, 300, 500 nm for PEA_2_PbBr_4_ single crystal (measured (points) and Gauss fitted (line) diffraction strain data as a function of sin^2^φ). The error bar indicates the standard deviation of the 2θ value. **e** The results of the layer-decomposed density of state calculation for PEA_2_PbI_4_ with and without (PEA^+^-I^−^) defect and schematics of the model structures vertically aligned with the layers.

To further confirm the structure difference between the surface and the interior of the 2D perovskites, we performed GIWAXS analysis and grazing-incidence X-ray diffraction (GIXRD) measurement with various incident angles. GIWAXS analysis with two different incident angles (α_c_ = 0.3° and 1°) was first conducted for PEA_2_PbI_4_. When the incident X-ray angle is 0.3°, the refraction spots are from the near-surface region of the crystal. On the other hand, the crystal structure close to the inside could be derived when the incident X-ray angle is 1°. The scattering patterns of PEA_2_PbI_4_ with a series of incident angles are shown in Fig. 4a. The sharp and discrete Bragg spots indicate that the crystal grains are highly oriented, with their (002) planes parallel to the substrate surface. As α_c_ increased to 1°, the diffraction patterns of PEA_2_PbI_4_ revealed similar patterns as that of near-surface. The GIWAXS profiles of two distinct incident angles are illustrated in Fig. 4b. When the incident angle increased from 0.3 to 1°, the locations of (004), (1$\bar{1}$0) and (1$\bar{1}$1) have slightly shifted to lower *q* values. Then we can derive the *d* spacings of (004), (1$\bar{1}$0) and (1$\bar{1}$1) by

2 $$d=2\pi /q$$

The lattice constants are a = 8.25 Å, b = 8.34 Å and c = 32.93 Å for the surface of the crystal; a = 8.35 Å, b = 8.43 Å, and c = 33.21 Å for the inside, demonstrating the compressive strain effect on the surface.

Then, we also measured XRD at more incident angles. Here PEA_2_PbBr_4_ is specially selected, which is also the scintillator material for later PET applications. As shown in Supplementary Fig. 7a, we measured XRD of PEA_2_PbBr_4_ at incident angles of 0.1°, 0.4°, 2.5°, 3°, 3.5°, 4°, 4.5°, and 5° respectively. As the angle increases, the location of (002) has slightly shifted to lower values, which represents an increase in the lattice constant. It is worth noting that the X-ray penetration depth increases upon enlarging the incidence angle. Therefore, the lattice structure information at different depths on the surface of the 2D perovskite single crystal can be obtained by varying the X-ray incident angle. The plot of estimated X-ray penetration depth *versus* incident angles for PEA_2_PbBr_4_ is shown in Supplementary Fig. 7b. The lattice constants are 8.0076 Å, 8.0152 Å, 8.0302 Å, 8.0454 Å, 8.0530 Å, 8.0606 Å, 8.0758 Å and 8.0988 Å respectively when the X-ray penetration depths are 3.8151 nm, 259.37 nm, 1896.5 nm, 2278.2 nm, 2659.7 nm, 3040.9 nm, 3422.0 nm and 3803.0 nm (Fig. 4c). Compared with the lattice constants calculated by single-crystal XRD result (8.31 Å), the strains are −3.64%, −3.55%, −3.37%, −3.18%, −3.09%, −3.00%, −2.82%, and −2.54% respectively. Moreover, we also employed the tilt-angle method to demonstrate the presence of the compressive strain on the two-dimensional perovskite surface. We picked up three representative depths of 100, 300 and 500 nm to check the residual strain in PEA_2_PbBr_4_ single crystals. The principle of this method could be found in previous studies^32^ and the detailed analysis of our results is documented in supporting information (Supplementary Fig. 8, Table 3). The positive slopes at both incident angles (Fig. 4d) also confirm the compressive strain at the surface of 2D crystals.

Density functional theory calculations were further carried out to verify the origin of the difference between surface and internal, using the VASP code^33,34^. Four distinct surface configurations were studied, including ${{{{{{\rm{PbI}}}}}}}_{4}^{2-}$, ${{{{{{\rm{PbI}}}}}}}_{2}$, ${{{{{{\rm{PEA}}}}}}}_{2}^{2+}$, and PEA^+^ exposed surfaces. Their surface energies are 0.213 J m^−2^, 3.036 J m^−2^, 0.491 J m^−2^, and 0.297 J m^−2^, respectively. Thus, the surface configuration with ${{{{{{\rm{PbI}}}}}}}_{4}^{2-}$ exposure is the most favorable one. As shown in Fig. 4e, using a slab model with surface ${{{{{{\rm{P}}}}}}{{{{{\rm{b}}}}}}{{{{{\rm{I}}}}}}}_{4}^{2-}$, we calculated the PEA_2_PbI_4_ band gap to be around 2.23 eV, using the self-energy corrected shell DFT-1/2 method^35–37^, with spin-orbit coupling considered (details given in Supplementary Note 2), consistent with experimental data of the bulk material (2.24 eV). Subsequently, we discuss the possibility of missing surface PEA^+^ ions since organic groups are usually more volatile. Because PEA^+^ is positively charged, its vacancy tends to emerge together with a surface I^−^ vacancy in order to meet charge neutrality. By introducing the PEA^+^-I^−^ co-vacancy complex on the surface, the a-b surface area of the supercell has contracted by ~2.2%. And the band gap increases to ~2.60 eV, which explains the short-wavelength emission in the PL spectra. It is noteworthy that such volume change is a spontaneous process that tends to release the stress induced by ion deficiency. The situation is quite distinct from forced compression. Indeed, perovskites like PEA_2_PbBr_4_ and PEA_2_PbI_4_ show reduced band gaps when subject to mechanical compression. An example of PEA_2_PbBr_4_ is given in Supplementary Fig. 9. As each lattice constant is scaled to 0.96 of the equilibrium one, the band gap is reduced by ~0.15 eV if the atomic coordinates are kept unchanged, and reduced by a less amount (0.12 eV) if octahedral distortion and rotation are permitted. It is known that distortion and tilting of the lead halide octahedron may reduce the overlap of electron cloud and lead to a higher band gap, but for these two perovskites, such gap enhancement is insufficient to compensate for the gap degradation due to lattice compression. Nevertheless, the lattice shrinkage in our materials is a consequence of surface ion missing, thus it is not a forced compression, but rather a spontaneous process for stress release. Note also that such lattice contraction is also important for band gap enhancement (the same rule is discovered for PEA_2_PbBr_4_, as shown in Supplementary Fig. 10). Going from the surface to the interior, the lattice shrinkage effect gradually goes away, rendering the band gap larger near the surface compared with the bulk. We also measured the XPS for the crystal to characterize the chemical difference between the surface and inside. The XPS N 1 *s* spectrum was initially recorded to show the surface states, and then the crystals were etched by Ar ions to expose the inside. As shown in Supplementary Fig. 11, the surface exhibits a negligible N 1 *s* peak. In contrast, the interior shows a typical peak at 402.3 eV of N 1 *s*. Such discrepancy confirms the presence of PEA^+^ vacancies on the surface.

**Fig. 5:**
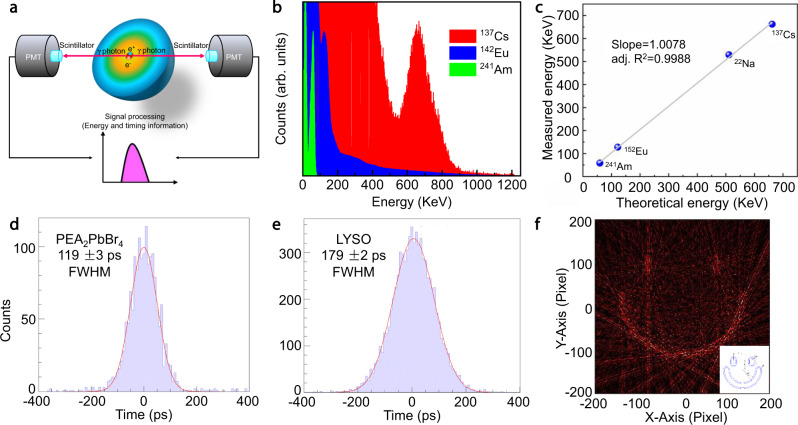
The γ-ray detection performance of 2D perovskite. **a** Schematic diagram of the energy and timing detection process. **b** Energy-resolved spectra of different radionuclide sources. **c** The high linear detection response of 2D perovskite scintillators to different radiation sources. **d, e** The measured 511 keV photopeak selection with resulting delay time histogram and Gaussian fit giving the CTR in FWHM of PEA_2_PbBr_4_ and LYSO. **f** The experimental imaging result of the smile face phantom filled with ^18^F-FDG sources in the channel.

Subsequently, we studied the γ-ray detection performance of these 2D perovskites and demonstrated the first image reconstruction of perovskites in PET application. In γ-ray detection (Fig. 5a), scintillators are usually coupled with a high-gain photomultiplier tube (PMT). Light output is one of the key parameters for scintillators. PEA_2_PbBr_4_ and BA_2_PbBr_4_ were excited by ^137^Cs γ-rays and the light output intensity (pulse height) was measured alongside LYSO single crystal (documented light yield: 33,200 photons/MeV) for reference (Supplementary Fig. 12a). PEA_2_PbBr_4_ and BA_2_PbBr_4_ exhibited peak channel number at 351 and 143 respectively. In comparison, LYSO showed a peak channel number at 327. We also corrected the photodetection efficiency of PMT toward three different samples according to the radioluminescence spectra of scintillators and the external quantum efficiency curve of PMT (Supplementary Fig. 12b). The γ-ray light yields of PEA_2_PbBr_4_ and BA_2_PbBr_4_ are calculated to be 38,800 photons/MeV and 17,400 photons/MeV. The light yield of PEA_2_PbBr_4_ is also much higher than previously reported perovskite scintillators (CsPbBr_3_ nanosheets, 21,000 photons/MeV^8^) and other composite-based scintillators with FRET mechanisms (gadolinium oxide–polymer nanocomposites, 27,000 photons/MeV^38^).

In Fig. 5b, the full-energy peaks of PEA_2_PbBr_4_ under irradiation by ^241^Am (59.5 keV), ^142^Eu (121 keV), and ^137^Cs (662 keV) could be clearly distinguished, and the resolved energy response with full-width at half maximum (FWHM) in percentage is 21.7% for 662 keV (^137^Cs γ-ray). The energy resolution is still inferior to conventional scintillators, like LYSO (8%) and LaBr_3_:Ce (3.5%)^39^. To determine the energy linearity of the 2D perovskite scintillator, we recorded an energy-calibrated spectrum according to the photopeak position of ^137^Cs γ-ray (662 keV). A series of photo peaks from different radionuclides were resolved at 59.5 keV (^241^Am γ-ray), 121 keV (^142^Eu γ-ray), 511 keV (^22^Na γ-ray), and 662 keV (^137^Cs γ-ray). As shown in Fig. 5c, the linear response is excellent with R^2^ as 0.9988. The superb linearity is attributed to the good optical transparency of two-dimensional perovskites. The reason is that γ-ray photons with different energies interact with the scintillators at different depths, and low optical transparency would lead to various light output efficiencies for different γ-ray sources. Moreover, the radiation stability of the 2D perovskites was evaluated (Supplementary Fig. 13). After radiation of 5040 Gy, XRD and PL of 2D perovskite PEA_2_PbBr_4_ remain basically unchanged.

We also studied the timing response and the possible application of 2D perovskites in PET. PET is a non-invasive molecular imaging technology with high biochemical sensitivity, ideal for the diagnosis and monitoring of cancer and tumors. In a PET system, a pair of photons are simultaneously produced by electron-positron annihilations, and emitted in opposite directions with the same energy. The location of the emission point along the line-of-response can be determined by the arrival time difference between the two annihilation photons, which is known as the time-of-flight (TOF) technique. The accuracy of the TOF difference is given by the coincidence time resolution (CTR). It should be noted that there are strict requirements of fast response speed for scintillators of PET, with the LYSO and LaBr_3_: Ce as numbered successful scintillators.

Here, the CTR value represents the timing response, and a smaller CTR value means faster response speed and higher accuracy as well as emission point localization precision in PET imaging applications. Since the CTR value depends on all the components of the detection chain (scintillator, photodetector, and performance of the digitizer/oscilloscope), we used the same setup to measure CTR from our perovskite scintillators and commercial LYSO crystals and compared them with LYSO crystals results in other literature^40–43^. It is worth noting that the crystal length is one of the main degradation factors of CTR. The CTR values and the absorption efficiency of gamma photons need to be weighed for practical use.

The CTR was measured with a home-built standard setup, and two back-to-back 511 keV γ-photons emitted by a ^22^Na source were detected from a pair of crystals. Si-PM was used as the photodetector. The energy spectra of the pair of scintillators are shown in Supplementary Fig. 14. The events between the two dashed lines are identified as the photo peak events, which are in the range of 400 keV to 600 keV. The time difference between the test detectors after applying a cut to select the photo peak is histogrammed in Fig. 5d, e. We refer to the FWHM of this distribution as the CTR value. We measured a CTR value of 103 ± 3 ps for a pair of 2 × 2 × 3 mm^3^ LYSO crystals and 179 ± 2 ps (Fig. 5e) for a pair of 2 × 2 × 20 mm^3^ LYSO crystals. As shown in Supplementary Table 4, for LYSO crystals of similar size, the CTR values measured by us are comparable to those reported in the literature, which verified the correctness of our facility. The CTR measured using two PEA_2_PbBr_4_ scintillators (4 × 4 × 0.8 mm^3^) is 119 ± 3 ps (Fig. 5d), showing considerable application potential in PET imaging. The improved CTR value is beneficial for achieving a higher spatial resolution along the line-of-response to diagnose smaller lesions and tumors. Moreover, we estimated the cost of PEA_2_PbBr_4_ as 2.09 RMB/g, much lower than LYSO (~15 RMB/g).

Furthermore, we demonstrated the possible applications of the perovskite scintillator in PET imaging. The small sensitive area of the single 4 × 4 mm^2^ Si-PM is not sufficient to build a prototype for imaging, thus we choose PMTs with larger sensitive areas and greater gain for our imaging experiment. In the prototype imaging experiment, only a pair of PEA_2_PbBr_4_ crystals were used, which might lower the imaging resolution. Figure 5f exhibits the imaging results of a smile face phantom, in which ^18^F-FDG were filled in the polyethylene channel. The redundant scatters are probably due to the detector cross talk, and further improvement relies on increasing the number of detectors and adding collimators. The geometric dimension of the phantom is shown in Supplementary Fig. 15. To the best of our knowledge, this is the first study showing the PET application of the emerging perovskite scintillators, demonstrating their excellent performance in fast timing response and high spatial resolution. It is worth noting that the thickness of the current 2D perovskite crystal is still not enough to reach high detection efficiency of 511 keV photons. Further improvement relies on the scalable fabrication of high-quality and large-size two-dimensional perovskite scintillators to achieve practical system performance evaluations. Another improvement is that the 2D perovskite sheet crystals could be aligned vertically toward the incident photons, i.e. the sheet plane is parallel to the incident direction of γ rays. Then the large length direction of the plane could be used to completely attenuate the photons. The advantage of this strategy is that we could preserve the thickness and the specific surface area, and thereby the SWS effect from the presence of crystal surface will not be weakened.

## Discussion

In summary, we posit the intrinsic strain in 2D perovskite crystals as a general phenomenon, which could be utilized to decrease the self-absorption effect without sacrificing the RL lifetime. Through combined experimental and theoretical study, the lacking of surface amine is verified as the structural origin of the surface compressive strain. The transient photoluminescence spectroscopy clearly reveals the fast energy transfer process from the crystal surface to inside bulks. The exemplary optical transparency and fast response speed enable their application in high-energy γ-ray detection. The energy response is highly linear with R^2^ as 0.9988, close to unity. On the practical side, we successfully demonstrated the first imaging reconstruction of source using a pair of 2D perovskite crystals as detectors in coincidence. The CTR value for the perovskite single crystals reached 119 ± 3 ps, which shows considerable application potential in PET imaging. This work provides a new paradigm for suppressing the self-absorption effect in perovskite scintillators and bridges the perovskite scintillators and practical hard X-ray as well as γ-ray detections.

## Methods

### Chemicals

Phenylethylamine bromide (PEABr, 99.9%), phenylethylamine iodide (PEAI, 99.9%), and n-Butylamine hydrobromide (BABr, 99.9%) were all purchased from Advanced Election Technology Co., Ltd. Lead bromide (PbBr_2_, 99.998%) and lead (II) iodide (PbI_2_, 99.999%) were purchased from Alfa Aesar. Hydrobromic acid (HBr, 40% wt/wt aq. sol.), N, N-Dimethylformamide (≥99.5%), acetone (≥99.5%), ethanol (75%), and isopropanol (≥99.7%) were all purchased from Sinopharm Group Co., Ltd. All the above reagents were used as received.

### Synthesis of two-dimensional perovskite single crystals

We used the seed-assisted volatile solvent method to grow two-dimensional perovskite single crystals. We dissolved PbBr_2_ and PEABr (1:2 molar ratio) in DMF at 25 °C under active mixing for 24 h to generate the 1.14 M PEA_2_PbBr_4_ solution. The solution was placed in a clean, smooth, and scratch-free Teflon beaker, which was sealed by tin foil with several open holes for solvent evaporation. Saturated solution and seed crystals were obtained by volatilizing slowly at 25 °C for several days. High-quality crystals were selected as seeds for further crystal growth. The volatilization speed is controlled by controlling the number of holes in the tin foil. The crystals were washed three times with isopropanol and stored in nitrogen. The same technique and seed-assisted volatile solvent method were adopted for crystallizing PEA_2_PbI_4_ and BA_2_PbBr_4_.

### Crystal characterization

PEA_2_PbBr_4_ SCs XRD measurements were performed using a Philips diffractometer (X pert pro-MRD) with a step of 0.017° and step time of 10.16 s. The lines used were Cu Kα_1_ and Cu Kα_2_ with wavelengths of 1.54060 Å and 1.54443 Å, respectively. The PL spectra were measured using a LabRAM HR800 with a He-Cd laser, with the excitation wavelength set to 370 nm for PEA_2_PbBr_4_. The absorption spectra were measured on an ultraviolet-visible spectrophotometer (PerkinElmer Instruments, Lambda 950) in transmission mode with an integrating sphere, which was calibrated by measuring a reference material (MgO powder) at the same time. The RL spectra in X-ray excitation were measured using an X-ray tube (M237, Newton Scientific) at 50 kV and a fluorescence spectrophotometer (FluoMax+, HORIBA). The time-resolved PL spectra were collected on an Edinburgh Instruments Ltd EPL-340. Excitation wavelengths were collected at 440 nm for PEA_2_PbBr_4_ SCs.

The time-resolved PL spectra in the wavelength domain were conducted on a home-built PL-scanned imaging microscope coupled with a time-correlated single photon counting (TCSPC) module to collect the PL kinetics.

GIXRD measurements were performed using a PANalytical B.V. diffractometer (X Pert Powder) with a step of 0.0052° and step time of 1.016 s. The lines used were Cu Kα_1_ and Cu Kα_2_ with wavelengths of 1.54060 Å and 1.54443 Å, respectively.

GIWAXS studies were performed at the BL16B1 beamline of the Shanghai Synchrotron Radiation Facility, Shanghai, China, using a beam energy of 12 keV (λ = 1.033 A˚) and a Mar 225 CCD detector. The grazing-incidence angles for the crystal were 0.3° and 1°, respectively. A GIXGUI Matlab toolbox was utilized for necessary corrections of GIWAXS raw patterns.

The pulse height spectrum system contains a PMT (HAMAMATSU R6231-100), scintillation preamplifier (ORTEC 113), spectroscopy amplifier (ORTEC 672), and multichannel analyzer (ORTEC ASPEC-927). The PMT was operated at a high voltage of 1000 V, and the pulse forming time of the multichannel analyzer was set to 0.5 microseconds. The pulse-height spectra of γ-ray were acquired through the same system, using ^137^Cs γ-ray source (662 keV, 10000 Bq), ^22^Na γ-ray source (511 keV, 5000 Bq), ^152^Εu γ-ray source (122 keV, 3500 Bq) and ^241^Am γ-ray source (59.5 keV, 400 Bq). All pulse height spectra were collected for more than ten minutes to ensure the formation of the full energy peak.

### CTR experiment settings

The dimension of the samples is 4 × 4 × 0.8 mm^3^ and wrapped by 5 layers of Teflon. Two 2 × 2 × 20 mm^3^ LYSO crystals were also tested for comparison. All test samples and crystals were coupled to a pair of 4 × 4 mm^2^ Si-PMs (HAMAMATSU S14160-4050HS) with optical glue (Dow corning RTV 3145) with single-ended readout.

All detectors were tested in a dark box with an ambient temperature at 18 °C. The distance between the front of two test detectors was 20 mm, while a ^22^Na radioactive source (activity of 9.53 μCi) was in the middle. The signals from the Si-PMs were discriminated by the NINO ASIC for timing measurement and also read out by the amplifier (AD8045) for energy measurement. All output signals were digitized in terms of a Tektronix DPO7254C oscilloscope.

### Imaging reconstruction experiment settings

The PET image reconstruction was achieved by rotating a pair of detectors with two crystals against each other. A pair of 2D perovskite crystals worked in the coincidence mode, which ensured that the collected data were generated by the solution. The crystals were optically coupled to Hamamatsu R2248 photomultiplier tubes (PMT) via the naturally plain bottom facets, while the top facets were covered in Teflon tape. All the PMTs and the coupled crystals were totally wrapped with black tape to avoid the effect of environment photons. The supply voltages of both PMTs were set to 1250 V, and the PMT outputs were directly connected to a Tektronics DPO 52108B digital storage oscilloscope with a 50 Ω input impedance. The oscilloscope was operated with a bandwidth of 2 GHz and a sampling rate of 6.25 Gsps per channel. One tubule of 1.2 mm inner diameter filled with the fludeoxyglucose ^18^F solution was used as the radioactive source. The coarse coincidence timing window width was set to 4 ns. The oscilloscope was triggered by an AND-logic event generated by the two PMT detectors, therefore ensuring that the majority of the resulting event pairs were coincidences. The trigger voltage was set to 30 mV to reduce false triggering. Each of the two pulses in a coincidence was sampled by the oscilloscope for 200 ns, resulting in 10000 data points. An energy window of 420 keV—600 keV was applied in energy discrimination.

The chosen experimental setup has the fundamental advantages of being easily reproducible while still allowing a full and direct comparison between the various digital time pickoffs under test.

## Supplementary information

Supplementary Information

## Data Availability

All data needed to evaluate the conclusions in the paper are present in the paper and in the Supplementary Information.
