# Supplementary material for: Self-wavelength shifting in two-dimensional perovskite for sensitive and fast gamma-ray detection
Source: Nat Commun. 2023 May 17;14:2808. doi: 10.1038/s41467-023-38545-y (PMC10192432; doi:10.1038/s41467-023-38545-y)
Supplement: Supplementary file 1 — Supplementary Information [file 41467_2023_38545_MOESM1_ESM.pdf]

SUPPLEMENTARY INFORMATION

Self-wavelength Shifting in Two-dimensional Perovskite for Sensitive and Fast Gamma-ray Detection

Jin et al.

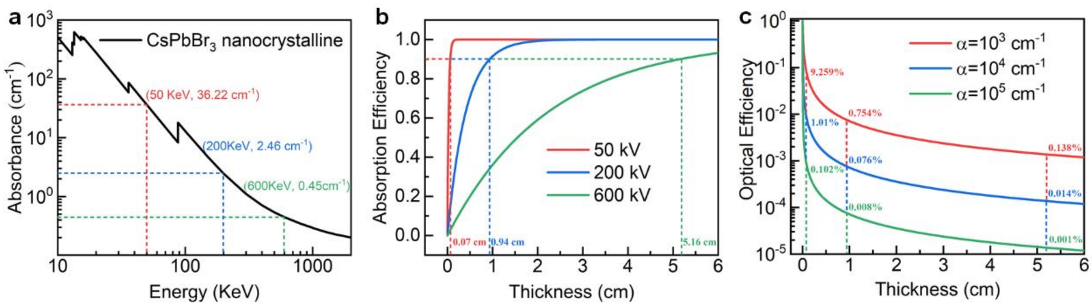

Supplementary Figure 1. The effect of thickness on X/γ-ray absorption and visible optical efficiency. (a) The absorption coefficient for X/γ-ray of $CsPbBr_3$ nanocrystalline as a function of photon energy. The photon cross section data (cm^2 g^{-1}) for different scintillators can be obtained from the following database: https://physics.nist.gov/PhysRefData/Xcom/html/xcom1.html. Then it is multiplied by the density (g cm^{-3}) of scintillators to obtain the attenuation coefficient (cm^{-1}) of scintillators to high-energy X/γ-rays. (b) Absorption efficiency for X/γ-ray as a function of thickness for different energies photons. (c) Optical efficiency as a function of thickness at different absorption coefficients.

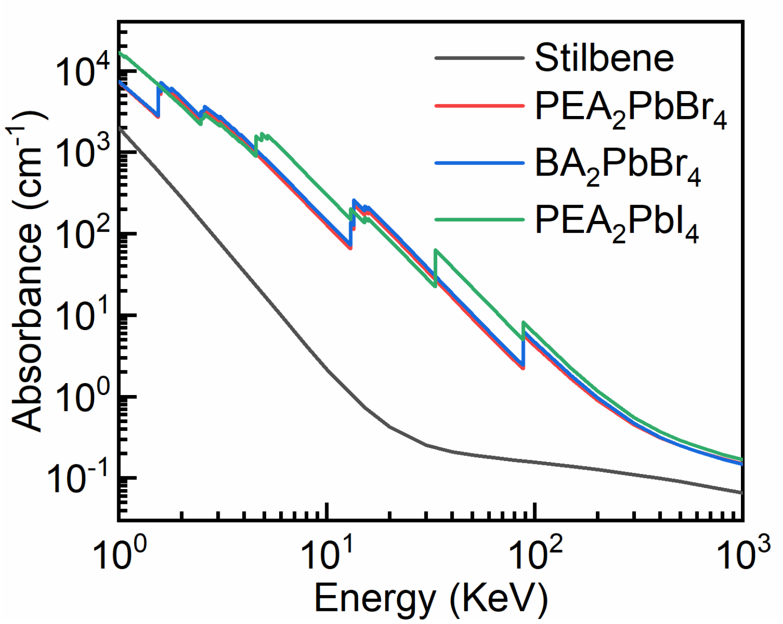

Supplementary Figure 2. The absorption coefficients as functions of photon energy for the two-dimensional perovskites. The photon cross section data ($cm^2\ g^{-1}$) for different scintillators can be obtained from the following database: https://physics.nist.gov/PhysRefData/Xcom/html/xcom1.html. Then it is multiplied by the density ($g\ cm^{-3}$) of scintillators to obtain the attenuation coefficient (cm^{-1}) of scintillators to high-energy X/γ rays..

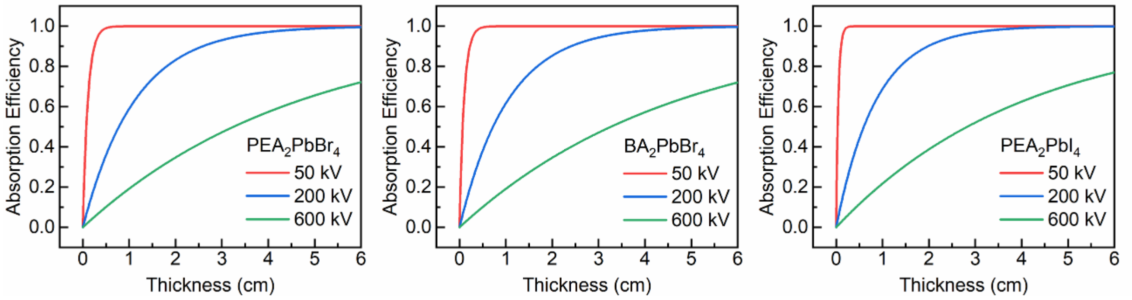

Supplementary Figure 3. The absorption efficiency as functions of thickness for the two-dimensional perovskites for different energy photons.

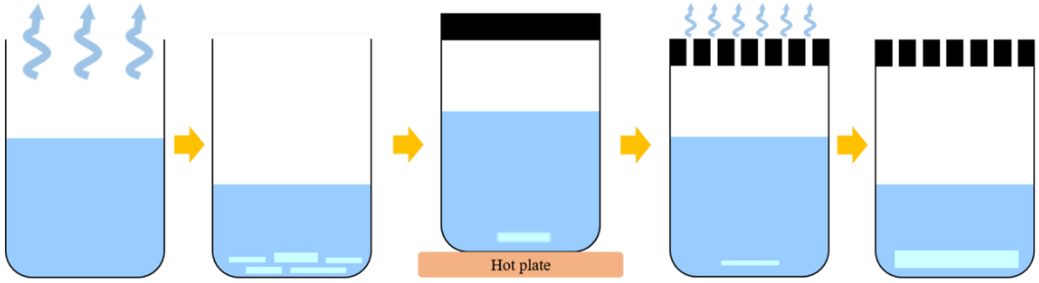

Supplementary Figure 4. Seed-assisted volatile solvent crystal growth process. $PbBr_2$ and PEABr (1:2 molar ratio) were dissolved in N, N-Dimethylformamide (DMF) at 25°C under active mixing for 24 h to generate the 1.14 M $(PEA)_2PbBr_4$ solution. The solution was placed in a clean, smooth, and scratch-free Teflon beaker, which was sealed by tin foil with several open holes for the solvent evaporation. Saturated solution and seed crystals were obtained by volatilizing slowly at 25 °C for several days. High-quality crystals were selected as seeds for further crystal growth. The same technique and seed-assisted volatile solvent method were adopted for crystallizing $(PEA)_2PbI_4$ and $(BA)_2PbBr_4$.

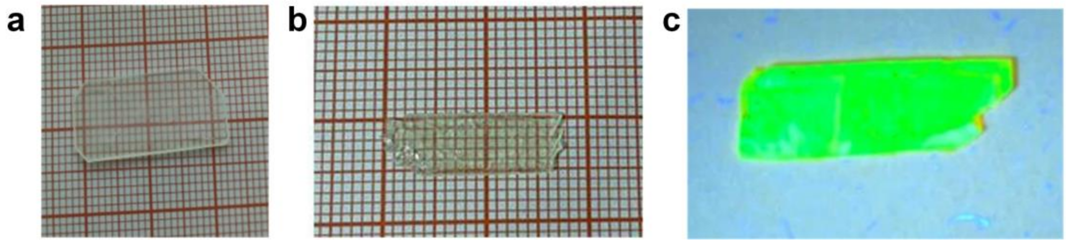

Supplementary Figure 5. Pictures of the synthesized single crystals. (a) PEA_2PbBr_4, (b) BA_2PbBr_4, (c) PEA_2PbI_4.

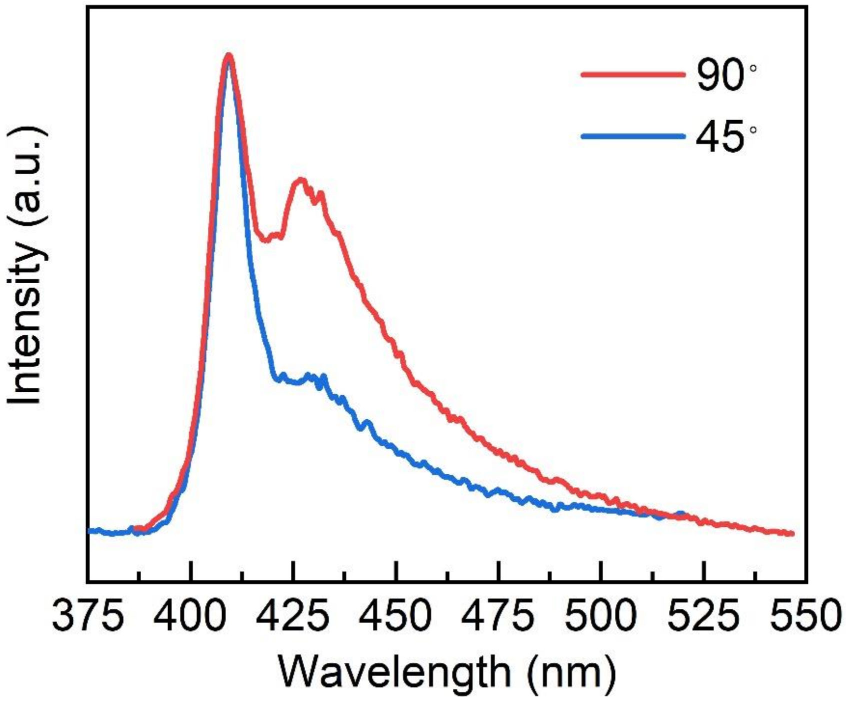

Supplementary Figure 6. Fluorescence spectra of photoluminescence excited at different incidence angles. The crystal used here is thinner than that in Figure 2.

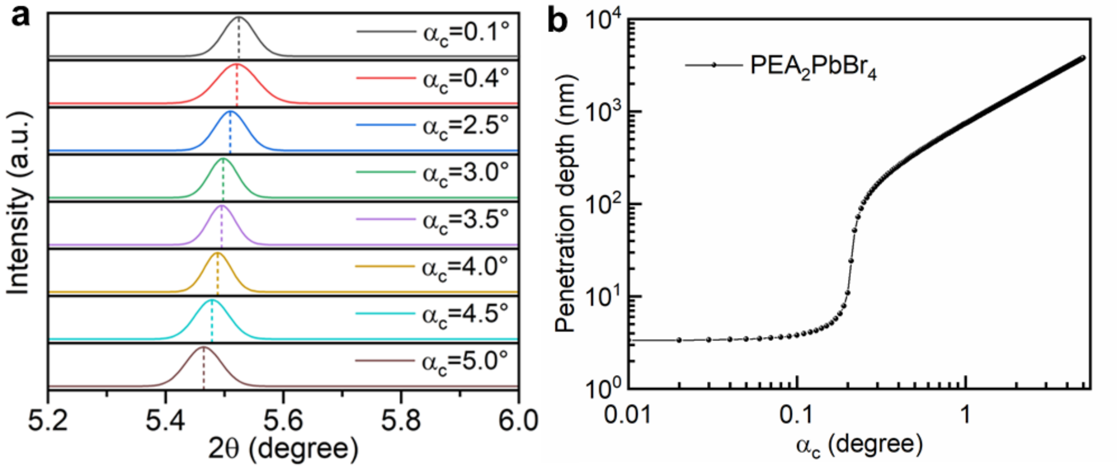

Supplementary Figure 7. (a) GIXRD spectra of PEA_2PbBr_4. (b) The plot of estimated X-ray penetration depth versus incident angles.

The X-ray penetration depth is estimated by the equation of

$$\tau(\alpha) = \frac{\sqrt{2}\lambda}{4\pi}\left\{\sqrt{(\alpha^2 - \alpha_c^2)^2 + 4\beta^2} - (\alpha^2 - \alpha_c^2)\right\}^{-1/2},$$

where λ is the wavelength of X-ray which is 1.033 Å in this work, α is the incident angle, α_c is the critical angle of the perovskite film, and β is the imaginary part that is related to the absorption coefficient. The critical angle α_c can be obtained by the equation of $\alpha_c = \sqrt{2\delta}$, while the values of δ and β can be calculated from the atomic scattering factors (f_1 and f_2) using:

$$\delta = \frac{n_a r_e \lambda^2}{2\pi} f_1$$

$$\beta = \frac{n_a r_e \lambda^2}{2\pi} f_2$$

where n_a is the number density, r_e is the classical electron radius of 2.818 $\times$ 10^{-15} m, and the atomic scattering factors (f_1 and f_2) of different elements can be obtained from the website 'https://henke.lbl.gov/optical_constants/asf.html'. The number density n_a is calculated by the equation of $n_a = \frac{\rho N_a}{M_a}$, where ρ is the physical density of perovskites (i.e. 2.27 g/cm3 for PEA_2PbBr_4), N_a is the Avogadro constant of 6.022 $\times$ 10^{23} mol^{-1}, and M_a is molar mass.

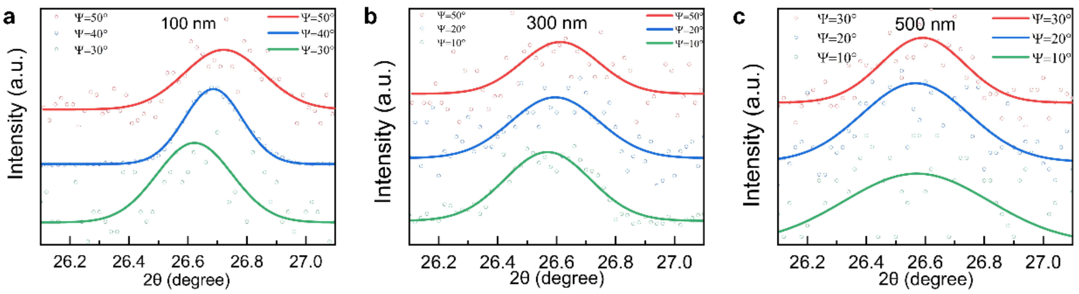

Supplementary Figure 8. GIXRD spectrum at different tilt angles at the depth of (a) 100 nm, (b) 300 nm and (c) 500 nm for PEA_2PbBr_4 single crystal.

The 2θ was fixed and the instrument angles (ω, ψ, ϕ) were varied according to the calculated parameters in **Supplementary Table 3** to obtain corresponding XRD patterns with penetration depth of 100 nm, 300 nm and 500 nm. The peak near 26.6° was chosen for further analysis. At each fixed depth, diffraction data were fitted with Gaussian distribution function to determine the peak location. The state of the residual stress and macroscopic residual strain can be judged by the slope k of $2\theta\text{-}\sin^2\varphi$ ($\varphi=\arccos(\cos\psi\cos(\omega-\theta))$) line. When $k < 0$, it is tensile strain/stress. When $k > 0$, it is compressive strain/stress, and the magnitude of strain/stress is determined by the value of the slope. As shown in Figure 4d, all fitting lines exhibited positive value in the slopes, and the slopes are 1.08 ± 0.045 @ 100nm, 0.09±0.013 @ 300nm and 0.07±0.084 @ 500nm respectively.

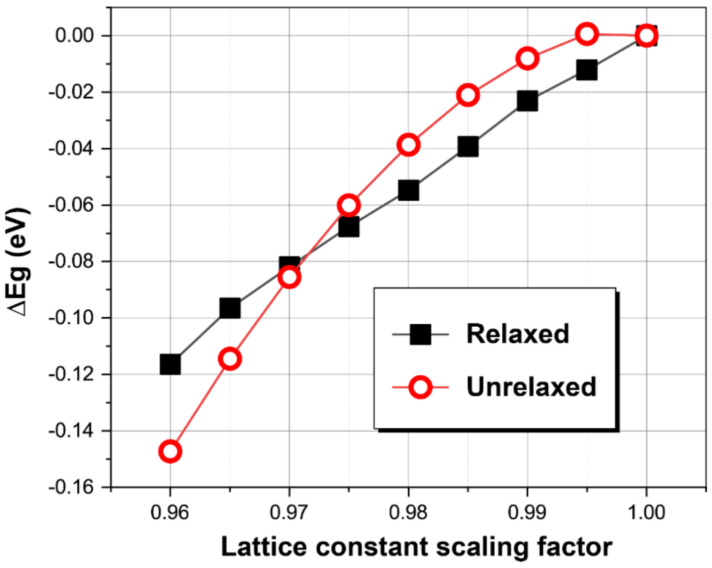

Supplementary Figure 9. Variation of PEA_2PbBr_4 band gap with respect to the lattice constant scaling factor during forced compression.

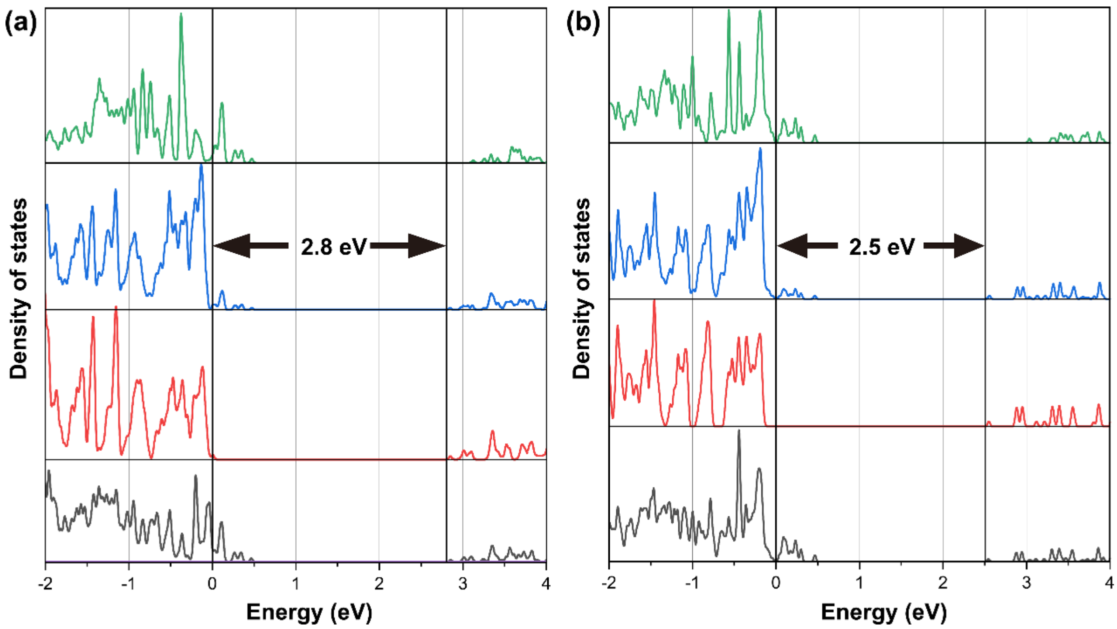

Supplementary Figure 10. Local density of states analysis for PEA_2PbBr_4 surface models. (a) With PEA^+ and I^- missing from the surface, allowing for lattice shrinkage; (b) No ion missing. The band gap of (b) deviates a bit from the calculated bulk value, because of the surface effect as well as the unavoidable structural arrangement during supercell construction.

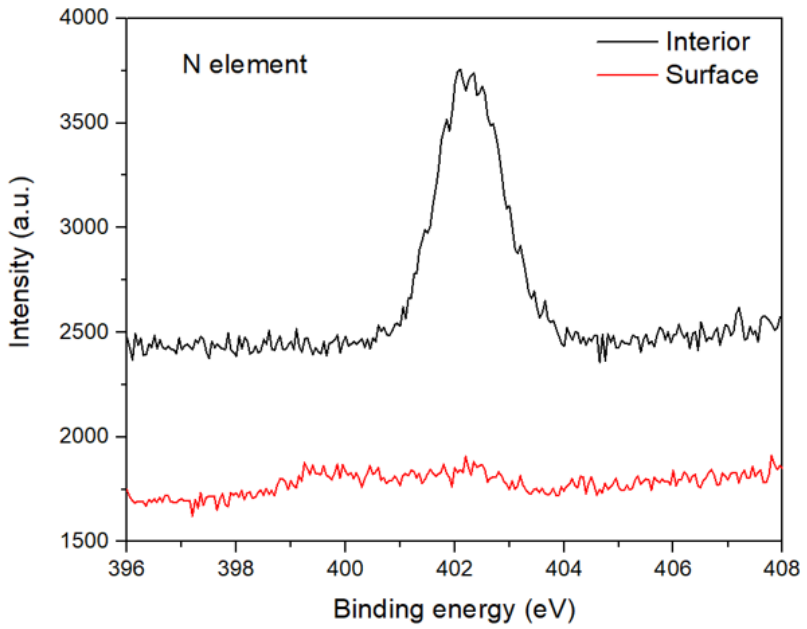

Supplementary Figure 11. N 1*s* XPS spectra of the surface and interior of two-dimensional perovskites PEA_2PbI_4.

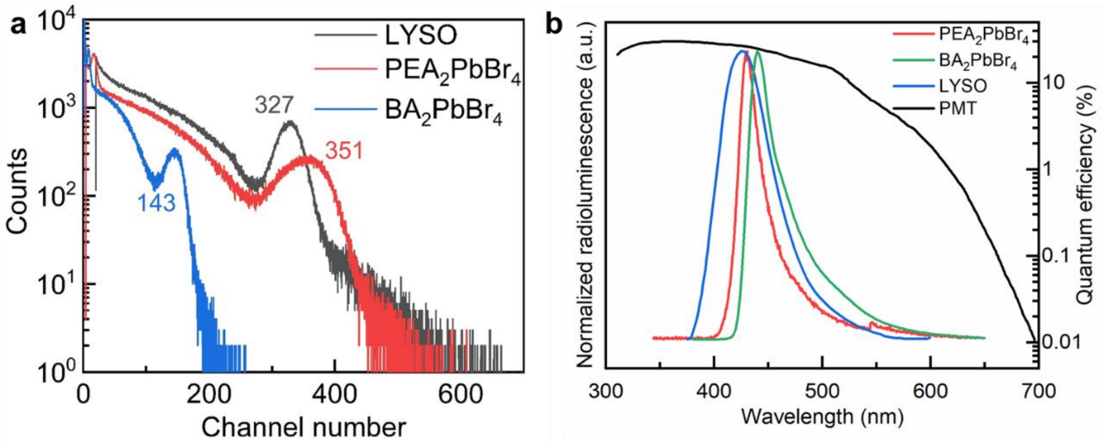

Supplementary Figure 12. Light yield measurement. (a) Pulse height spectrum of LYSO plastic scintillator, PEA_2PbBr_4 and BA_2PbBr_4 single crystals under irradiation by ^{137}Cs γ-ray. (b) The normalized radioluminescence spectra (red line for PEA_2PbBr_4, green line for BA_2PbBr_4 and blue line for LYSO) and the quantum efficiency curve (black line) for the photomultiplier tube. The normalized detection efficiency toward LYSO, PEA_2PbBr_4 and BA_2PbBr_4 is 24%, 22% and 20% respectively.

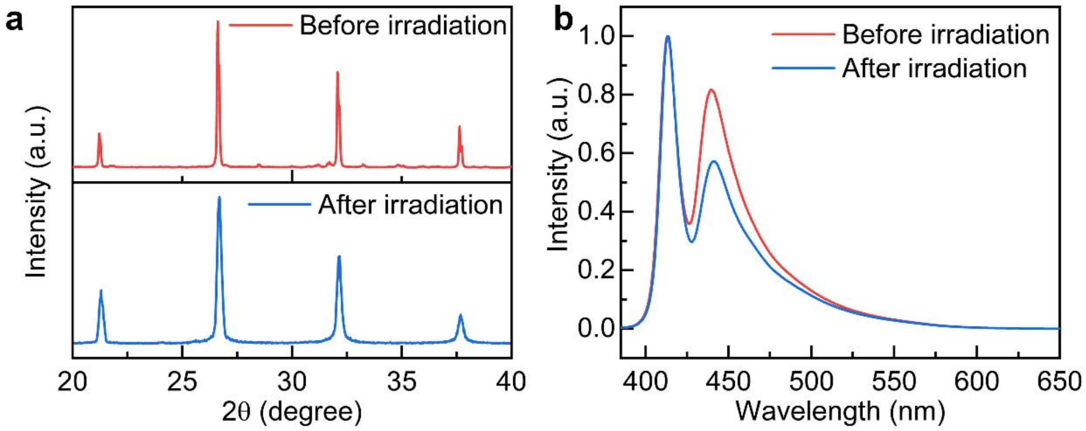

Supplementary Figure 13. Stability test of PEA_2PbBr_4. (a) XRD of PEA_2PbBr_4 before and after radiation. (b) PL of PEA_2PbBr_4 before and after radiation. The PEA_2PbBr_4 single crystal without encapsulation was irradiated for 2.8 hours by a ^{60}Co isotope radioactive source with a dose rate of 1800 Gy/h, and the cumulative irradiation dose was about 5040 Gy.

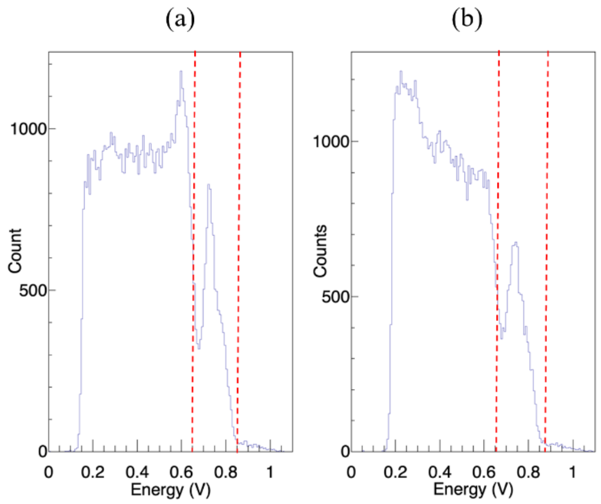

Supplementary Figure 14. Energy spectra of (a) sample 1 of PEA_2PbBr_4, (b) sample 2 of PEA_2PbBr_4.

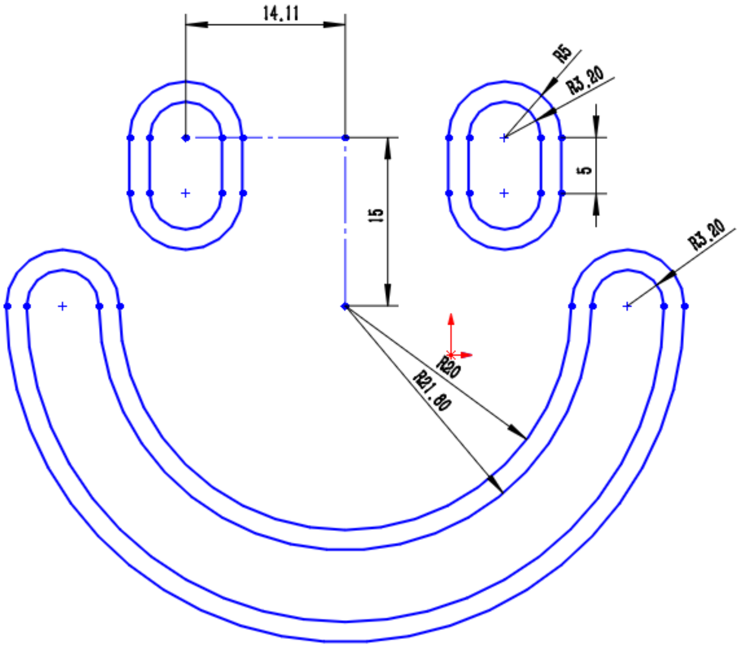

Supplementary Figure 15. The geometric dimension of the phantom. The units in the figure are millimeters.

Supplementary Table 1. Optical transparency and luminescence lifetime of different types of perovskite scintillators.

Material	Optical transparency	Luminescence lifetime	Ref.
$CsPbBr_3$ QD	48.9%	7 ns	7
$CsPbBr_3$@Cs_4PbBr_6	62.0%	3 ns	9
$MAPbBr_3$ QD	75.1%	17.4 ns	23
$FAPbBr_3$ QD	69.1%	18.7 ns	24
$RuCuBr_3$	95.5%	41400 ns	14
$Cs_3Cu_2I_5$	98.5%	968 ns	15
$Cs_5Cu_3Cl_6I_2$	99.8%	40000 ns	16
$Cs_2Ag_{0.6}Na_{0.4}In_{0.85}Bi_{0.15}Cl_6$	99.5%	1300 ns	17
Ru_2CuCl_3	94.4%	12210 ns	18
K_2CuCl_3	99.9%	12970 ns	19
PEA_2PbBr_4	87.6%	6.28 ns	This work
PEA_2PbI_4	87.0%	0.49 ns	This work
BA_2PbBr_4	95.8%	4.93 ns	This work

Supplementary Table 2. The fitting results of the time-dependent photoluminescence spectra for the two-dimensional perovskites.

Material	τ_1	τ_2	Adj-R^2
PEA_2PbBr4	4.4±0.15 ns (75.1%)	11.9±1.54 ns (24.9%)	0.998
BA_2PbBr4	3.6±0.03 ns (89.2%)	14.4±0.69 ns (10.8%)	0.999
PEA_2PbI4	0.37±0.01 ns (96.6%)	6.58±0.5 ns (3.4%)	0.996

Supplementary Table 3. GIXRD test (Angle setting, 2θ and slope fitting results).

Penetration depth (nm)	ψ (degree)	ω (degree)	ϕ (degree)	2θ (degree)	slope
100	30	0.6225	-65.76	26.62±0.022	1.08±0.045
	40	0.7061	-70.82	26.68±0.011	
	50	0.8463	-73.91	26.72±0.013	
300	10	1.717	-40.25	26.57±0.007	0.09±0.013
	20	1.806	-59.24	26.59±0.023	
	50	2.747	-76.32	26.62±0.029	
500	10	3.024	-43.74	26.57±0.045	0.07±0.084
	20	3.193	-62.45	26.56±0.021	
	30	3.514	-70.95	26.59±0.014	

Supplementary Table 4. Comparison of CTR values of different single crystals scintillator with different sizes.

Material	Size (mm^3)	CTR value (ps)	Reference
LYSO	3 × 3 × 5	200	40
	3 × 3 × 15	230	40
	3 × 3 × 5	186	41
	3 × 3 × 15	220	41
	2 × 2 × 3	103 ± 3	This work
	2 × 2 × 20	179 ± 2	This work
LYSO: Ce	2 × 2 × 10	136.4~230.5	42
	2 × 2× 3	69 ± 3	43
PEA_2PbBr_4	4 × 4 × 0.8	119 ± 3	This work

Supplementary Note 1. Definition of absorption efficiency and optical efficiency.

The absorption efficiency of high-energy photons is defined as

$$\eta_{abs} = 1 - e^{-\alpha d},$$

where e is the natural log, α is the absorption coefficient for X/γ photons and d is the thickness.

The optical efficiency represents the ratio of outgoing light to pristine light after passing through a specific distance within the scintillator, which is defined as

$$\eta_{efficiency} = \frac{I}{I_0} = e^{-\alpha d},$$

where I_0 is incident light intensity, I is outgoing light intensity, e is the natural log, α is the absorption coefficient and d is the thickness.

Supplementary Note 2. Electronic structure calculations of PEA_2PbI_4 and PEA_2PbBr_4.

Density functional theory (DFT) suffers from a well-known problem in recovering the fundamental gap of semiconductors and insulators, at least under its local density approximation (LDA) or generalized gradient approximation (GGA) forms. One explanation is that these approximations only use local or semi-local forms for the electron exchange, and the incorrect exchange energy mainly leads to insufficient cancellation of the spurious electronic self-interaction. Including these unphysical self-interactions tend to over-estimate the levels of the valence band, reducing the band gap. Hybrid functionals are indeed more reasonable approaches, but the high computational load forbids their applications in very large supercells such as PEA_2PbI_4 and PEA_2PbBr_4 surface models with a certain amount of organic cations and inorganic anions missing, especially considering that spin-orbit coupling has to be taken into account simultaneously. Hence, in this work the electronic structure calculations require an efficient method that is yet accurate in terms of the band gaps.

There is an efficient self-energy correction method named DFT-1/2, proposed in 2008 by Ferreira and coworkers. The method introduces some self-energy potentials, obtained from atomic calculations, onto those anions that contribute to the valence band. The self-energy potentials have to be trimmed by a spherical cutoff function, otherwise the overlapping of -1/r tails would render the total energy divergent. The selected cutoff radius should lead to a maximized band gap, thus it is not an empirical parameter, but rather should be obtained in a variational way. An improved form of DFT-1/2 was further given in 2018, named shell DFT-1/2, which aims at better fitting covalent semiconductor calculations. Yet, in the mean time, in certain cases it may improve the electronic structures for ionic insulators and semiconductors, especially when large anions are present. The shell DFT-1/2 method involves two cutoff radii for the self-energy potential, an outer cutoff radius (r_{out}) as well as inner cutoff radius (r_{in}), both should be optimized to maximize the band gap. Whenever $r_{in} = 0$, shell DFT-1/2 automatically reduces to conventional DFT-1/2, but for large anions one usually finds a fairly large r_{in}. In this work, Br^- and I^- are large anions. Hence, shell DFT-1/2 is adopted for electronic structure calculations, with non-collinear DFT runs that consider spin-orbit coupling.

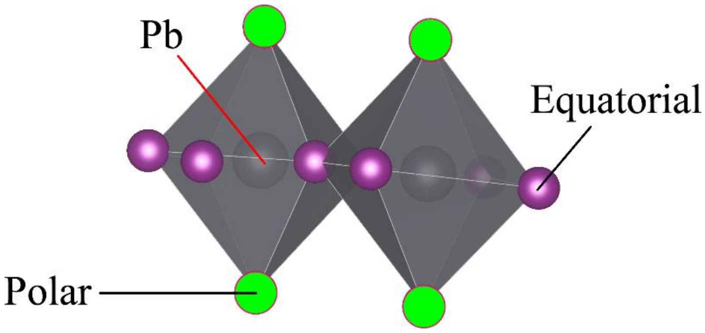

In these perovskite materials, the only anion is the halogen elements (Br or I). However, their structures are quite complicated, and the same halogen element appears in very distinct chemical

environments. For simple semiconductors, the optimization of r_{out}/r_{in} may be simultaneously done for all anions of a specific element just once. However, in a strict sense, the cutoff radii should be optimal for each anion individually, according to our previous work (RSC Advances 7, 21856 (2017)). Taking PEA2PbI4 as an example, the I anions can be roughly divided into equatorial ones ($I^{(1)}$) and polar ones ($I^{(2)}$). They are subject to quite different chemical environments. Hence, to obtain more accurate electronic structures, we optimize the self-energy potential cutoff radii for $I^{(1)}$ and $I^{(2)}$ separately. The optimized values are r_{in} = 1 bohr, r_{out} = 3.1 bohr for $I^{(1)}$; while r_{in} = 1.5 bohr, r_{out} = 2.9 bohr for $I^{(2)}$. Shell DFT-1/2 band gap calculated at these optimal cutoff radii is 2.20 eV, with spin-orbit coupling considered.

For PEA_2PbBr_4, the same phenomenon is observed. The optimized cutoff radii are r_{in} = 0 bohr, r_{out} = 2.5 bohr for $Br^{(1)}$; while r_{in} = 1.2 bohr, r_{out} = 2.2 bohr for $Br^{(2)}$. Shell DFT-1/2 band gap calculated at these optimal cutoff radii is 2.73 eV, with spin-orbit coupling considered.
